# Supplementary material for: Mild Heat Stress Alters the Physical State and Structure of Membranes in Triacylglycerol-Deficient Fission Yeast, Schizosaccharomyces pombe
Source: Cells. 2024 Sep 13;13(18):1543. doi: 10.3390/cells13181543 (PMC11430649; doi:10.3390/cells13181543)
Supplement: Supplementary file 1 [file cells-13-01543-s001.zip › cells-3179698-supplementary.pdf]

cells

MDPI

Supplementary Material

Mild heat stress alters the physical state and structure of membranes in triacylglycerol-deficient fission yeast, *Schizosaccharomyces pombe*

Péter Gudmann[1,2], Imre Gombos[1], Mária Péter[1], Gábor Balogh[1], Zsolt Török[1], László Vígh[1], Attila Glatz[1,*]

1 Biological Research Centre, Institute of Biochemistry, HUN-REN, 6726 Szeged, Hungary; gudmann.peter@brc.hu (P.G.); gombos.imre@brc.hu (I.G.); peter.maria@brc.hu (M.P.); balogh.gabor@brc.hu (G.B.); torok.zsolt@brc.hu (Z.T.); vigh.laszlo@brc.hu (L.V.); glatz.attila@brc.hu (A.G.)
2 Doctoral School of Environmental Sciences, University of Szeged, 6720 Szeged, Hungary
* Correspondence: glatz.attila@brc.hu (A.G.)

Table S1. Fluorescence intensity of the mitochondrial network in WT and DKO cells after staining with MitoTracker Red CMXRos. (n = 3 independent experiments; mean ± SD)

Strain	Temperature	Fluorescence Intensity
WT	30°C	0.0249+/-0.0010
	40°C 1 h	0.0297+/-0.0014
DKO	30°C	0.0245+/-0.0026
	40°C 1 h	0.0278+/-0.0015

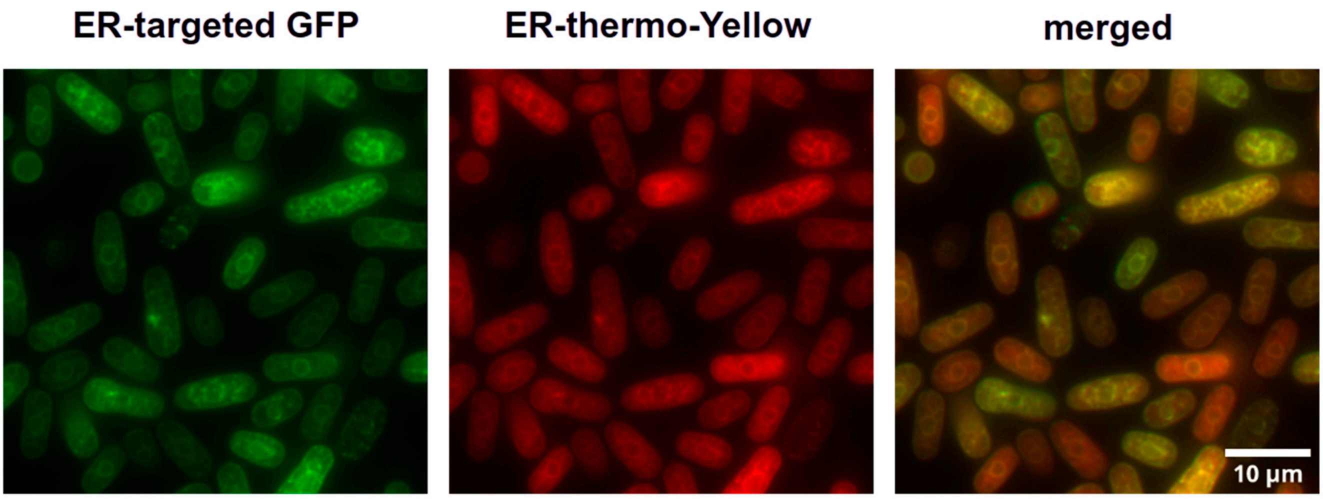

Figure S1. ER-thermo-yellow is localized in the cortical endoplasmatic reticulum. Images from left to right: BRC98 strain (carrying ER-targeted GFP); BRC98 cells stained with ER-thermo-yellow; merged image.

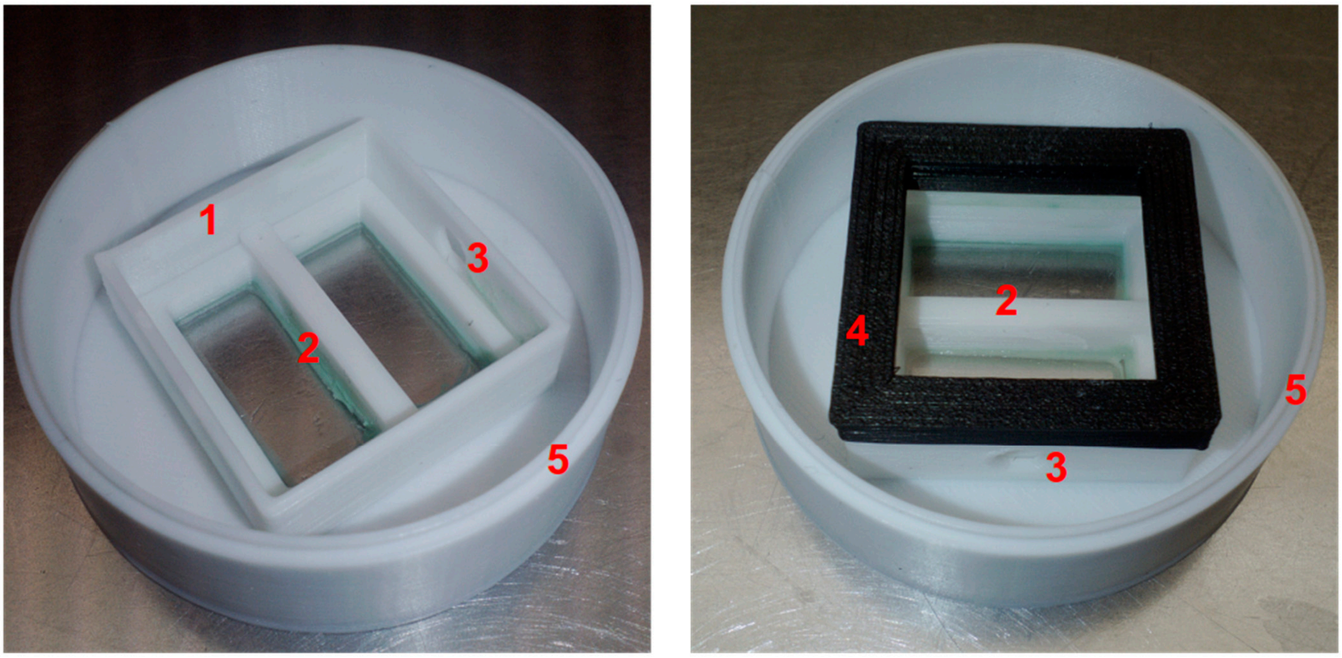

Figure S2. Our 3D-printed sample holder for GP measurements was designed to fit into the 35 mm wide sample holder unit of the Leica TCS SP5 confocal microscope. Main parts: chamber (1) with a glass lid (4) to prevent heat loss and evaporation during the heat-shock experiment; dual slot sample holder (2) enabling sample measurements in parallel at the same temperature (+/-0.5°C); hole (3) for thermometer to measure the exact chamber temperature; stackable side (5) for storing multiple holders.
